# Random forest can accurately predict the technique failure of peritoneal dialysis associated peritonitis patients

**DOI:** 10.3389/fmed.2023.1335232

**Published:** 2024-01-17

**Authors:** Zhiyun Zang, Qijiang Xu, Xueli Zhou, Niya Ma, Li Pu, Yi Tang, Zi Li

**Affiliations:** ^1^Department of Nephrology, Institute of Nephrology, West China Hospital of Sichuan University, Chengdu, China; ^2^Department of Nephrology, Yibin Second People's Hospital, Yibin, China

**Keywords:** peritoneal dialysis, peritonitis, technique failure, machine learning algorithms, prediction model

## Abstract

**Instructions:**

Peritoneal dialysis associated peritonitis (PDAP) is a major cause of technique failure in peritoneal dialysis (PD) patients. The purpose of this study is to construct risk prediction models by multiple machine learning (ML) algorithms and select the best one to predict technique failure in PDAP patients accurately.

**Methods:**

This retrospective cohort study included maintenance PD patients in our center from January 1, 2010 to December 31, 2021. The risk prediction models for technique failure were constructed based on five ML algorithms: random forest (RF), the least absolute shrinkage and selection operator (LASSO), decision tree, k nearest neighbor (KNN), and logistic regression (LR). The internal validation was conducted in the test cohort.

**Results:**

Five hundred and eight episodes of peritonitis were included in this study. The technique failure accounted for 26.38%, and the mortality rate was 4.53%. There were resignificant statistical differences between technique failure group and technique survival group in multiple baseline characteristics. The RF prediction model is the best able to predict the technique failure in PDAP patients, with the accuracy of 93.70% and area under curve (AUC) of 0.916. The sensitivity and specificity of this model was 96.67 and 86.49%, respectively.

**Conclusion:**

RF prediction model could accurately predict the technique failure of PDAP patients, which demonstrated excellent predictive performance and may assist in clinical decision making.

## Introduction

Peritoneal dialysis (PD) is a preferred and cost-effective renal replacement therapy for end-stage renal disease (ESRD) patients (1, 2). In the context of the global prevalence of coronavirus disease 2019 (COVID-19), PD is more advantageous than hemodialysis (HD) because of its home-based use (3, 4). However, peritoneal dialysis associated peritonitis (PDAP) is one of the common but severe complications in the process of PD (5), which greatly damages the peritoneal function and limits the use of PD (6). Overall peritonitis rate is 0.06–1.66 episodes per patient-year (7, 8), which is a significant reason for catheter dysfunction events, repeated hospitalization and technique failure (9). PDAP directly contributes to 22% catheter removal, 18% permanent transfer to HD, and 2–6% death (10), accounting the most important complication that leads to technique failure. The outcomes of peritonitis varied markedly among different centers and countries despite the guidelines of PDAP (11). Therefore, identifying the high-risk population of technique failure has important clinical value for improving the management and prognosis of PDAP patients.

Machine learning (ML) algorithms are important bioinformatics research methods in the clinic, which can process a large number of clinical datasets and incorporate multiple indicators to construct prediction models to assess the risk of outcomes (12). In the PDAP population, the previous study constructed a model which showed excellent prediction performance for pathogen-specific diagnosis based on ML algorithms (13). Some studies also attempted to predict the prognosis of PDAP patients using ML algorithms. One single-center study constructed a nomogram by using multivariate COX regression, which could predict cardiovascular events in PDAP patients (14). There are also studies attempting to predict treatment failure in PDAP patients (15–18). However, these studies only included a portion of clinical variables to screen predictors, which may ignore important variables. Meanwhile, these studies only used logistic regression (LR) to construct prediction models, without constructing multiple models and comparing their performances. In addition, some of the above studies did not establish independent test datasets to validate the performance of risk prediction tool (17, 18).

With incorporating all possible clinical variables, we intended to construct multiple prediction models based on ML algorithms, aiming to find the best prediction model to predict the technique failure in PDAP patients, which was helpful to identify high-risk patients, optimize the management of PDAP patients, and improve their prognosis.

## Methods

### Study population

This retrospective study enrolled the maintenance PD patients who were hospitalized due to PDAP in our PD center of the West China Hospital, Sichuan University, from January 1, 2010 to December 31, 2021.

All PDAP patients were diagnosed according to the criteria of 2022 International Society for Peritoneal Dialysis (ISPD) guidelines (4). The details are as follows: PDAP should be diagnosed when at least two of the following are present: (1) clinical features consistent with peritonitis (abdominal pain and/or cloudy dialysis effluent); (2) dialysis effluent white cell count >100 × 10^6^/L, with >50% polymorphonuclear leukocytes; (3) positive dialysis effluent culture.

The patients included herein were chosen according to the following criteria: (1) maintenance peritoneal dialysis patients admitted to our department due to PDAP, and (2) age ≥ 18 years old. The exclusion criteria were as follows: (1) episodes with PD < 3 months, (2) episodes with recovered renal function, and (3) episodes with missing data (absence of the peritoneal dialysate white cell count or the results of pathogenic culture).

This study was approved by the Medical Ethics Committee of West China Hospital of Sichuan University, Sichuan, China. This study was registered at the Thai Clinical Trials Registry (TCTR20180313004). This study followed the Declaration of Helsinki, and written informed consents were obtained from all participants.

### Data collection

The following clinical and laboratory data were collected from the medical records and laboratory information system. The demographic variables, including age, gender, height, weight, systolic blood pressure (SBP) and diastolic blood pressure (DBP) were obtained. The body mass index (BMI) was calculated based on height and weight. The patients’ comorbidities were recorded, including diabetes mellitus (DM), cirrhosis, cardiovascular diseases (CVDs), connective tissue diseases, etc. The CVDs were defined as the presence of coronary heart disease, heart failure, stroke, and peripheral arterial disease (19). The Charlson Comorbidity Index Score (CCI) was calculated based on the patients’ age and comorbidities (20). PD modality [continuous ambulatory peritoneal dialysis (CAPD) or automated peritoneal dialysis (APD)] and PD duration were also recorded. The laboratory variables included hemoglobin (HB), white blood cell (WBC), serum albumin (ALB), serum creatinine (SCr), blood urea nitrogen (BUN), uric acid (UA), estimated glomerular filtration rate (eGFR), triglycerides (TG), high-density lipoprotein cholesterol (HDL-C), low-density lipoprotein cholesterol (LDL-C), serum potassium (K), serum calcium (Ca), N-terminal pro-brain natriuretic peptide (NT-proBNP), high-sensitive C-reactive protein (hs-CRP), fibrinogen (FIB), ferritin, etc. And eGFR was calculated by the Chronic Kidney Disease Epidemiology Collaboration (CKD-EPI) equation. The peritoneal dialysate white cell counts on day 1, day 3 and day 5 were collected. The results of causative organisms and the regimens of initial intraperitoneal (IP) antibiotic were recorded.

### Outcomes

The patients’ outcomes were technique failure and technique survival. Technique failure was defined as PD catheter removal, transfer to HD (temporary or permanent) or peritonitis-related death (21). Peritonitis-related death included death within 4 weeks after the onset of PDAP, death with active peritonitis, or any death during hospitalization for a peritonitis episode. And technique survival was defined as complete resolution of peritonitis without the need for PD catheter removal (21). Complete resolution was defined as the white cell counts <100/uL in peritoneal dialysates with a relief of clinical manifestations.

### Data process

Before the statistical analysis and construction of prediction models, we conducted the data preprocessing. The raw data was verified and checked independently by two authors (ZZY and XQJ). To improve the quality and reliability of the data, we have corrected the errors, omissions, duplicates, or outliers in the raw data. We transformed the data consistently and reproducibly in order to be recognized by R software. To reduce statistical workload and variables unrelated to the outcomes of PDAP patients, univariate analysis was utilized to identify all possible variables. Variables with *p* value < 0.1 in the univariate analysis were selected to construct the prediction models. Data with less than 15% missing values were imputed by mean or median (22). Data with more than 15% missing values were excluded from the final analysis.

### Statistical analysis

Data normality was analyzed using the Kolmogorov–Smirnov test. Normal distribution data were expressed by mean ± standard deviation (SD), while non-normal distribution data were represented by median and interquartile range (IQR). Count data were expressed as the number of cases (%). Continuous variables were analyzed by Student’s t test or Mann–Whitney U test, while categorical variables were analyzed by Chi-square test or Fisher’s exact test. The IBM SPSS Statistics version 26.0 (IBM Corp., Armonk, NY, United States) and R software version 4.2.2 (R Foundation, Vienna, Austria) were used to perform the statistical analyses. A two-sided *p* value less than 0.05 was considered statistically significant.

### Construction and validation of prediction models based on ML algorithms

Seventy-five percent of all patients were randomly allocated to the training cohort and constructed a prediction model to predict the technique failure of PDAP patients, while the remaining 25% were assigned to the test cohort for internal validation. The optimal hyperparameters were selected from 5-fold cross-validation in the training cohort. The performance of prediction models was evaluated by accuracy, sensitivity, specificity, precision, recall, F1-score and area under curve (AUC). The confusion matrix and receiver operating characteristic curves (ROC) were presented in the analysis.

In this study, five machine learning algorithms were applied to construct the prediction models of technique failure in PDAP patients, including random forest (RF), the least absolute shrinkage and selection operator (LASSO), decision tree, k nearest neighbor (KNN), and LR.

## Results

**Figure 1:**
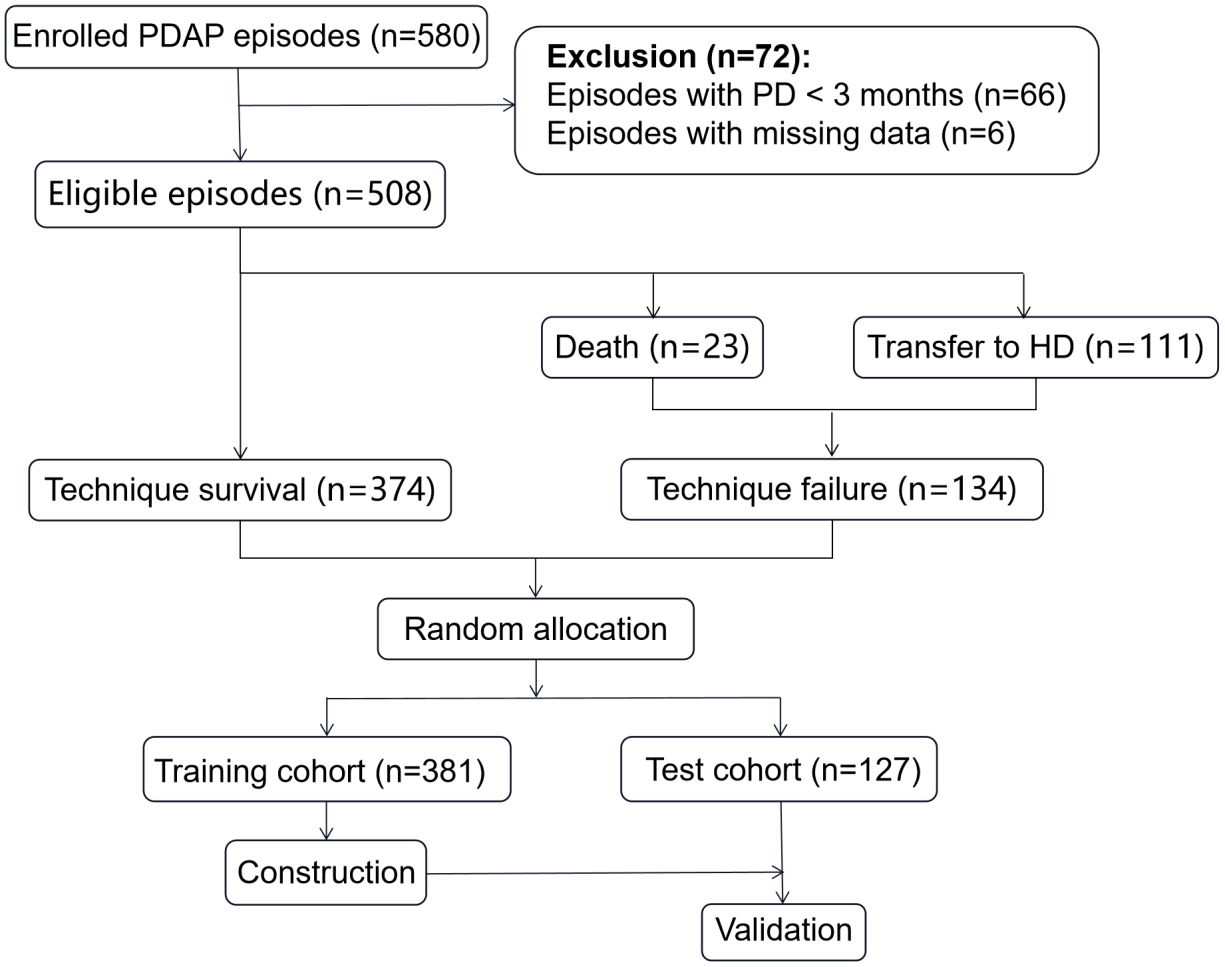
Flow chart for the study participants enrollment and outcomes.

The flow chart of this study is presented in Figure 1. A total of 580 PDAP episodes were enrolled in our study. However, 72 episodes were excluded: 66 episodes with PD less than 3 months, and 6 episodes missing the peritoneal dialysate white cell count. No patients had recovered renal function.

### Characteristics of study participants

There were 508 eligible episodes in 413 PDAP patients in the current study. Figure 1 shows the clinical outcomes of the PDAP patients. The technique failure accounted for 26.38%, and the mortality rate was 4.53% (23 episodes). Switching from PD to HD was the leading reason (111 episodes, 21.85%) of technique failure. And technique survival was achieved in 374 (73.62%) episodes.

The baseline characteristics of all patients are shown in Table 1. The median age was 50.0 years old and the male patients accounted for 53.0%. The median PD duration was 25.2 months and CAPD was the main PD modality (97.4%). Among the eligible patients, 85 patients (16.7%) had DM, with 116 patients (22.8%) having CVDs, and the median score of CCI was 4 points.

**Table 1:** Baseline demographic characteristics.

Variables	Total (*n* = 508)	Technique failure group (*n* = 134)	Technique survival group (*n* = 374)	*p* value^*^
Male, *n*%	269 (53.0)	72 (53.7)	197 (52.7)	0.841
Age, year, IQR	50.0 (41.0, 60.0)	50.0 (42.0, 63.0)	49.5 (40.0, 60.0)	0.389
BMI, kg/m^2^, IQR	22.9 (20.4, 26.0)	22.8 (19.5, 24.7)	22.9 (20.8, 26.4)	0.123
PD duration, months, IQR	25.2 (12.2, 51.2)	37.2 (14.2, 73.2)	24.9 (10.9, 48.0)	**0.001**
DM, *n*%	85 (16.7)	23 (17.2)	62 (16.6)	0.893
CVD, *n*%	116 (22.8)	41 (30.6)	75 (20.1)	**0.016**
CCI score, points, IQR	4 (3, 5)	4 (3, 6)	4 (3, 5)	**0.022**
SBP, mmHg, IQR	135 (120, 150)	135 (121, 153)	135 (120, 150)	0.907
DBP, mmHg, ± SD	84 ± 16	86 ± 17	83 ± 16	0.855
APD, *n*%	13 (2.6)	2 (1.5)	11 (2.9)	0.529
History of kidney transplantation, *n*%	6 (1.2)	3 (2.2)	3 (0.8)	0.198
History of HD, *n*%	104 (20.5)	30 (22.4)	74 (19.8)	0.534

*Comparison between technique failure group and technique survival group. The bold values were *p* values < 0.05.

The details of glycolipid metabolism, electrolyte, coagulation and inflammatory variables are displayed in Table 2. The PDAP related tests and initial IP antibiotic regimens are displayed in Table 3. There were 255 culture-positive episodes (50.2%) in all PDAP patients. And coagulase-negative Staphylococcus was the leading bacteria (18.3%) in the causative organisms.

**Table 2:** Baseline characteristics of laboratory variables.

Variables	Total (*n* = 508)	Technique failure group (*n* = 134)	Technique survival group (*n* = 374)	*p* value^*^
HB, g/L, IQR	93 (76, 105)	89 (76, 102)	96 (77, 107)	**0.019**
PLT, 10^9^/L, IQR	192 (142, 262)	220 (160, 292)	180 (136, 243)	**0.001**
WBC, 10^9^/L, IQR	7.06 (5.65, 9.64)	7.89 (6.19, 11.00)	6.83 (5.34, 9.24)	**0.001**
Neutrophils, 10^9^/L, IQR	5.55 (4.01, 7.73)	6.27 (4.85, 9.28)	5.17 (3.87, 7.50)	**0.000**
DB, μmol/L, IQR	1.8 (1.3, 2.6)	2.0 (1.3, 3.0)	1.8 (1.3, 2.5)	0.212
IB, μmol/L, IQR	2.9 (2.0, 4.3)	2.3 (1.6, 3.7)	3.1 (2.2, 4.5)	**0.000**
ALT, IU/L, IQR	11.0 (7.0, 18.0)	9.0 (5.0, 17.0)	12.0 (8.0, 19.0)	**0.001**
AST, IU/L, IQR	17.0 (13.0, 23.0)	16.0 (12.0, 26.0)	18.0 (13.0, 23.0)	0.273
ALB, g/L, ± SD	29.6 ± 6.2	26.3 ± 6.2	30.6 ± 5.9	**0.000**
GLB, g/L, IQR	27.7 (23.9, 31.6)	29.2 (24.3, 33.7)	27.1 (23.8, 30.9)	**0.003**
A/G ratio, ± SD	1.10 ± 0.30	0.91 ± 0.29	1.15 ± 0.29	**0.000**
BUN, mmol/L, IQR	17.2 (13.0, 21.6)	16.2 (11.6, 20.8)	17.5 (13.5, 22.1)	**0.025**
SCr, μmol/L, IQR	840.5 (650.1, 1045.2)	817.0 (650.0, 1048.0)	847.0 (650.8, 1046.0)	0.608
eGFR, ml/ (min*1.73m^2^), IQR	5.08 (4.18, 6.64)	5.22 (4.28, 6.59)	5.01 (4.13, 6.64)	0.484
Cys-C, mg/L, IQR	5.98 (5.03, 7.08)	6.09 (5.31, 7.17)	5.92 (4.92, 7.04)	0.094
UA, μmol/L, IQR	342.0 (298.0, 395.3)	337.0 (292.1, 401.8)	343.5 (298.0, 394.3)	0.711
Carbon dioxide binding force, mmol/L, IQR	25.9 (23.4, 28.3)	25.5 (22.5, 27.9)	26.2 (23.6, 28.5)	**0.015**
iPTH, pmol/L, IQR	20.49 (8.76, 35.67)	21.86 (11.25, 40.51)	19.14 (7.85, 34.15)	0.121
NT-proBNP, pg./mL, IQR	7,458 (4,217, 17,692)	17,692 (12,278, 19,936)	6,059 (3,705, 10,348)	**0.022**
GLU, mmol/L, IQR	5.55 (4.62, 7.12)	5.74 (4.80, 7.51)	5.49 (4.60, 6.99)	0.185
TG, mmol/L, IQR	1.29 (0.91, 1.90)	1.46 (1.03, 2.06)	1.26 (0.89, 1.77)	**0.035**
CHOL, mmol/L, IQR	4.0 (3.30, 4.63)	3.62 (3.10, 4.28)	4.08 (3.44, 4.78)	**0.000**
HDL-C, mmol/L, IQR	1.10 (0.83, 1.39)	0.93 (0.68, 1.23)	1.15 (0.90, 1.43)	**0.000**
LDL-C, mmol/L, IQR	2.13 (1.67, 2.73)	1.90 (1.38, 2.33)	2.21 (1.76, 2.81)	**0.000**
Na, mmol/L, ± SD	138.5 ± 4.2	137.0 ± 4.2	138.9 ± 4.1	**0.000**
K, mmol/L, IQR	3.75 (3.33, 4.23)	3.65 (3.23, 4.11)	3.77 (3.34, 4.29)	0.080
Cl, mmol/L, IQR	95.7 (91.9, 99.9)	94.5 (91.2, 98.5)	96.0 (92.1, 100.2)	**0.006**
Mg, mmol/L, IQR	0.82 (0.71, 0.98)	0.79 (0.68, 0.97)	0.82 (0.72, 0.98)	**0.033**
Ca, mmol/L, IQR	2.17 (2.02, 2.29)	2.16 (1.98, 2.28)	2.17 (2.02, 2.29)	0.189
P, mmol/L, IQR	1.37 (1.08, 1.74)	1.40 (1.06, 1.78)	1.36 (1.08, 1.72)	0.773
PT, s, IQR	12.1 (11.4, 13.0)	12.4 (11.6, 13.7)	12.0 (11.3, 12.9)	**0.003**
INR, IQR	1.06 (1.00, 1.15)	1.10 (1.03, 1.20)	1.05 (1.00, 1.12)	**0.000**
APTT, s, IQR	30.0 (26.3, 34.2)	31.3 (27.6, 37.9)	29.3 (25.7, 33.1)	**0.000**
FIB, g/L, IQR	5.09 (4.34, 6.46)	5.56 (4.81, 6.75)	4.90 (4.23, 6.04)	**0.000**
Ferritin, ng/mL, IQR	260.8 (139.6, 501.7)	322.3 (180.5, 640.5)	243.7 (125.4, 459.9)	**0.003**
IL-6, pg./mL, IQR	27.65 (10.05, 91.00)	68.61 (17.41, 135.50)	22.73 (9.49, 75.59)	**0.012**
hs-CRP, mg/L, IQR	47.5 (9.8, 111.0)	131.0 (37.6, 187.5)	33.8 (8.4, 90.0)	**0.000**
PCT, ng/mL, IQR	2.03 (0.65, 10.64)	2.03 (0.65, 9.62)	2.03 (0.64, 11.53)	0.583

*Comparison between technique failure group and technique survival group. The bold values were *p* values < 0.05.

**Table 3:** Baseline characteristics of peritoneal dialysate and IP antibiotic regimens.

Variables	Total (*n* = 508)	Technique failure group (*n* = 134)	Technique survival group (*n* = 374)	*p* value^*^
**Peritoneal dialysate white cell counts**				
Day 1, 10^6^/L, IQR	1,165 (276, 3,895)	990 (260, 3,200)	1,250 (285, 4,100)	0.351
Day 3, 10^6^/L, IQR	120 (70, 545)	545 (343, 2,105)	120 (40, 213)	**0.000**
Day 5, 10^6^/L, IQR	30 (20, 270)	270 (230, 303)	20 (18, 60)	**0.000**
**Causative organisms**				
Culture negative peritonitis, *n*%	255 (50.2)	54 (40.3)	201 (53.7)	**0.009**
*Staphylococcus aureus* peritonitis, *n*%	26 (5.1)	11 (8.2)	15 (4.0)	0.068
Streptococcal peritonitis, *n*%	22 (4.3)	0 (0.0)	22 (5.9)	**0.002**
Coagulase-negative Staphylococcus peritonitis, *n*%	93 (18.3)	21 (15.7)	72 (19.3)	0.435
Corynebacterium peritonitis, *n*%	18 (3.5)	5 (3.7)	13 (3.5)	1.000
Enterococcus peritonitis, *n*%	14 (2.8)	4 (3.1)	10 (2.7)	0.768
Pseudomonas peritonitis, *n*%	8 (1.6)	1 (0.7)	7 (1.9)	0.687
Acinetobacter peritonitis, *n*%	9 (1.8)	1 (0.7)	8 (2.1)	0.457
Fungal peritonitis, *n*%	25 (4.9)	21 (15.7)	4 (1.1)	**0.000**
Polymicrobial peritonitis, *n*%	6 (1.2)	5 (3.7)	1 (0.3)	**0.006**
Enteric gram-negative bacteria peritonitis, *n*%	32 (6.3)	11 (8.2)	21 (5.5)	0.303
**Initial IP antibiotic regimens**				
First-generation cephalosporin + third-generation cephalosporin, *n*%	364 (71.7)	88 (65.7)	276 (73.8)	0.075
First-generation cephalosporin + aminoglycosides, *n*%	41 (8.1)	11 (8.2)	30 (8.0)	1.000
Third-generation cephalosporin + vancomycin, *n*%	61 (12.0)	17 (12.7)	44 (11.8)	0.759
Vancomycin + aminoglycosides, *n*%	19 (3.7)	8 (6.0)	11 (2.9)	0.118
Quinolones + vancomycin, *n*%	23 (4.5)	10 (7.4)	13 (3.5)	0.086

*Comparison between technique failure group and technique survival group. The bold values were *p* values < 0.05.

### Comparison between technique failure group and technique survival group

In order to explore the potential predictors, we conducted univariate analysis (Tables 1–3). Table 1 showed there were no significant statistical differences in gender, age, BMI, SBP, DBP and PD modality. However, the PD duration of the technique failure group was significantly longer than the technique survival group (37.2 months vs. 24.9 months, *p* = 0.001). In complications, we found that higher incidence of CVDs and higher scores of CCI in the technique failure group (*p* = 0.016 and *p* = 0.022, respectively).

As shown in Table 2, there were statistical differences between the two groups in many laboratory variables such as ALB, NT-proBNP, HDL-C, FIB, ferritin, etc.

The peritoneal dialysate white cell counts in the technique failure group on day 3 (545 × 10^6^/L vs. 120 × 10^6^/L, *p* < 0.001) and on day 5 (270 × 10^6^/L vs. 20 × 10^6^/L, *p* < 0.001) were significantly higher than the technique survival group (Table 3). There was no statistical difference in the initial IP antibiotic treatment regimens between the two groups (*p* = 0.143), and the PDAP patients received first-generation cephalosporin plus third-generation cephalosporin frequently (Table 3).

With regard to the results of causative organisms (Table 3), there was a significantly lower rate of culture-negative episodes between the failure group and the survival group (40.3% vs. 53.7%, *p* = 0.009). There was also a significant statistical difference in the composition of causative organism spectrum between the two groups (*p* < 0.001). The main bacteria of failure group were fungi (15.7%) and coagulase-negative Staphylococcus (15.7%). While the technique survival group was mainly composed of coagulase-negative Staphylococcus (19.3%), followed by Streptococcus (5.9%).

### Construction and validation of prediction models based on ML algorithms

**Figure 2:**
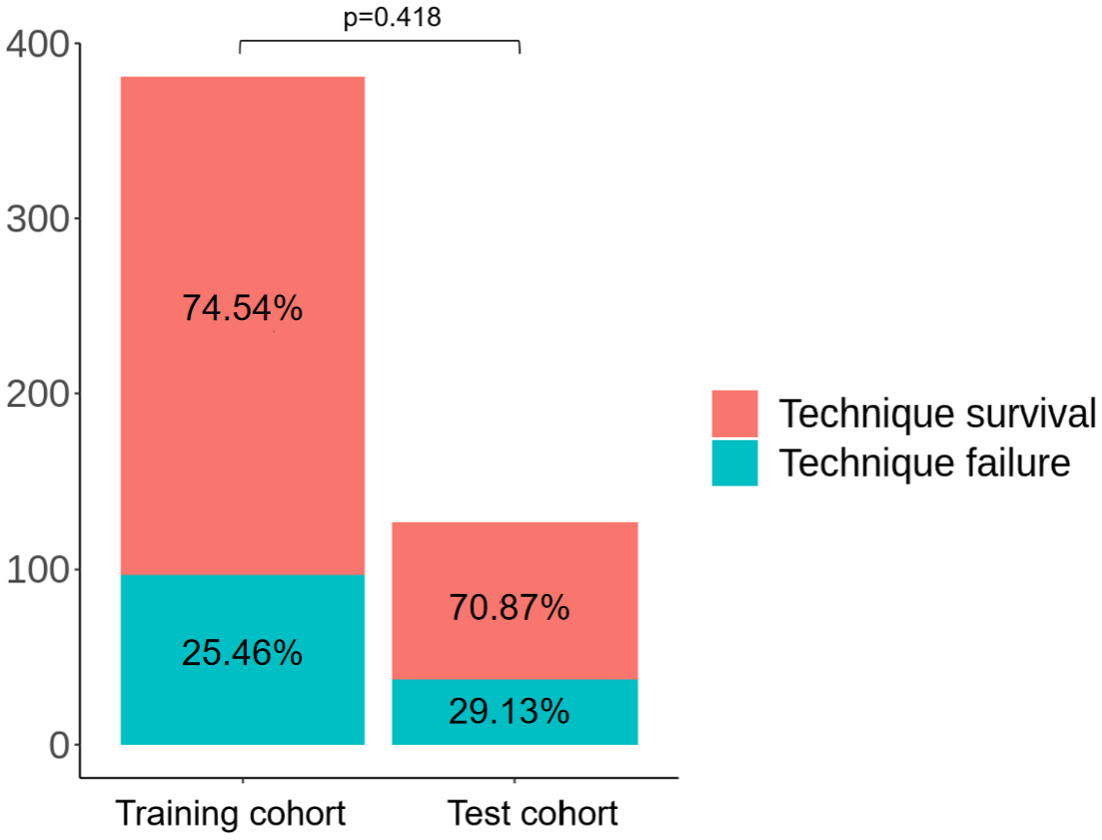
Comparison of clinical outcomes between training cohort and test cohort.

We preprocessed the data before the construction of prediction models. We deleted some variables with high missing rates, such as IL-6, hs-CRP, and PCT. Then, we included variables with *p* values less than 0.05 and interpolated the missing values by the mean or median for the construction of prediction models. The patients were randomly divided into training cohort and test cohort according to the ratio of 0.75 to 0.25. There was no statistical difference in the incidence of technique failure between the training cohort and the test cohort (25.46% vs. 29.13%, *p* = 0.418, Figure 2), indicating that the random allocation was feasible.

**Figure 3:**
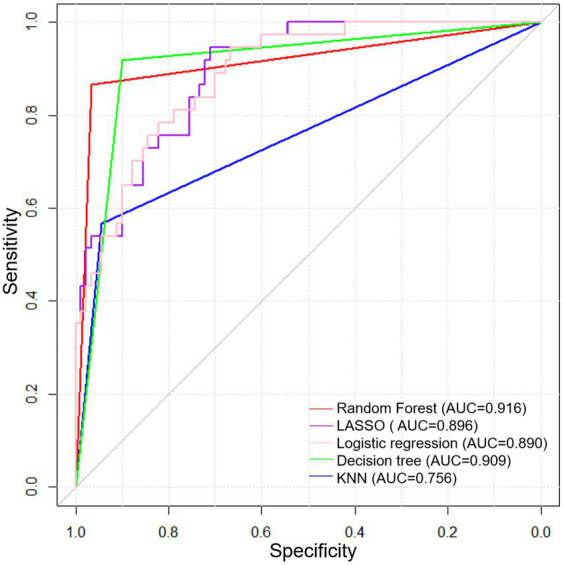
ROC curves of prediction models based on machine learning algorithms.

We then constructed the prediction models for technique failure in PDAP patients based on LR, LASSO, RF, KNN and decision tree. Figure 3 displays the ROC curves and Table 4 shows the validation of prediction models. Our results demonstrated that the RF model had excellent performance in predicting technique failure in PDAP patients compared with other prediction models, which showed the highest AUC value (0.916).

**Table 4:** Validation of the prediction models.

Prediction models	Accuracy	Sensitivity	Specificity	Precision	Recall	F1-score
LR	74.80%	94.59%	66.67%	53.85%	94.59%	0.6863
LASSO	83.46%	51.35%	96.67%	86.36%	51.35%	0.6440
RF	93.70%	96.67%	86.49%	91.43%	96.67%	0.9398
Decision tree	90.55%	90.00%	91.89%	79.07%	90.00%	0.8418
KNN	83.46%	56.76%	94.44%	80.77%	56.76%	0.6667

**Figure 4:**
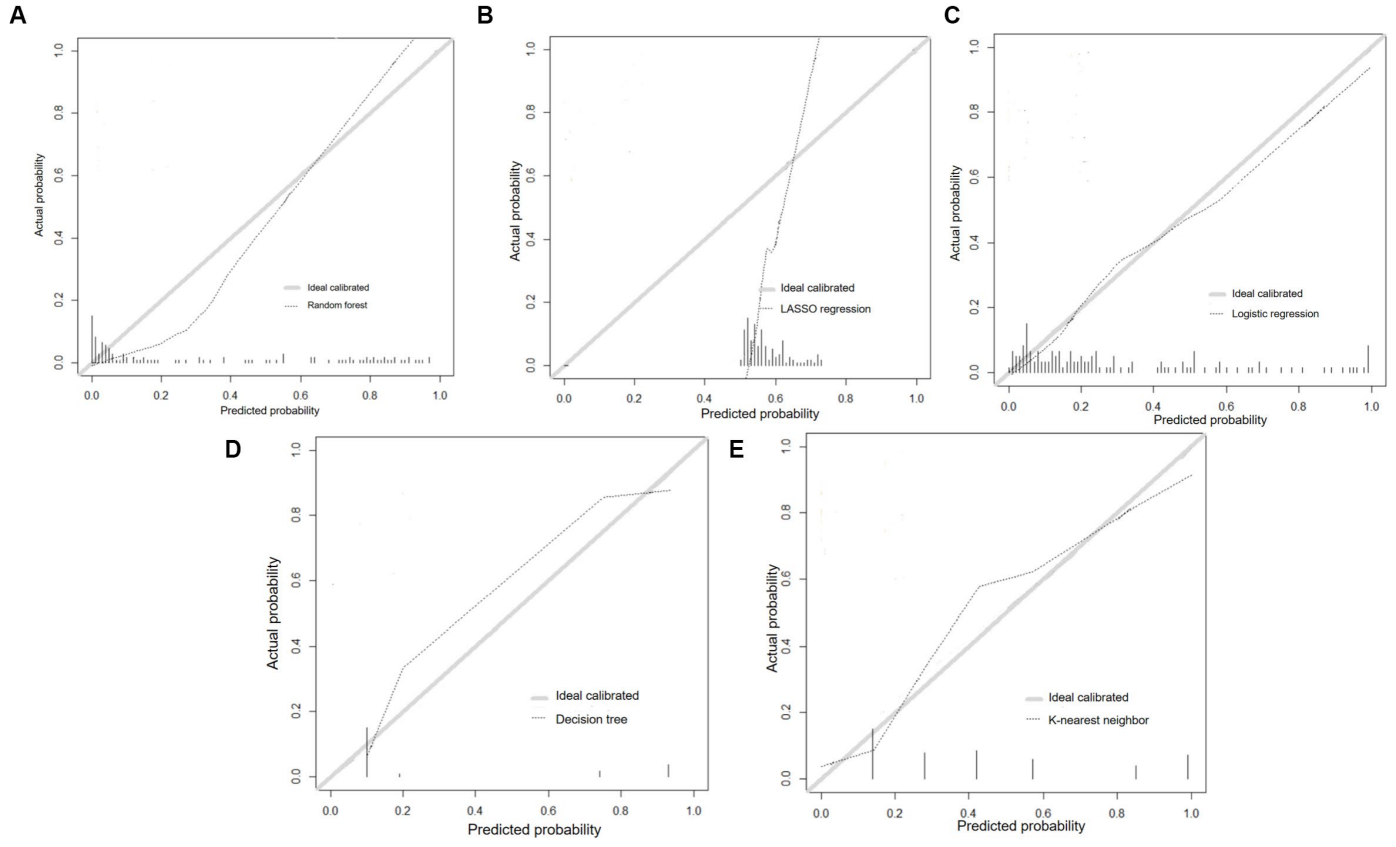
Calibration curves of prediction models. **(A)** Random forest; **(B)** LASSO regression; **(C)** Logistic regression; **(D)** Decision tree; **(E)** K-nearest neighbor.

The calibration curves of prediction models were depicted in Figures 4A–E, and the LR and RF models were close to perfectly calibrated line which demonstrated favorable calibration ability.

**Figure 5:**
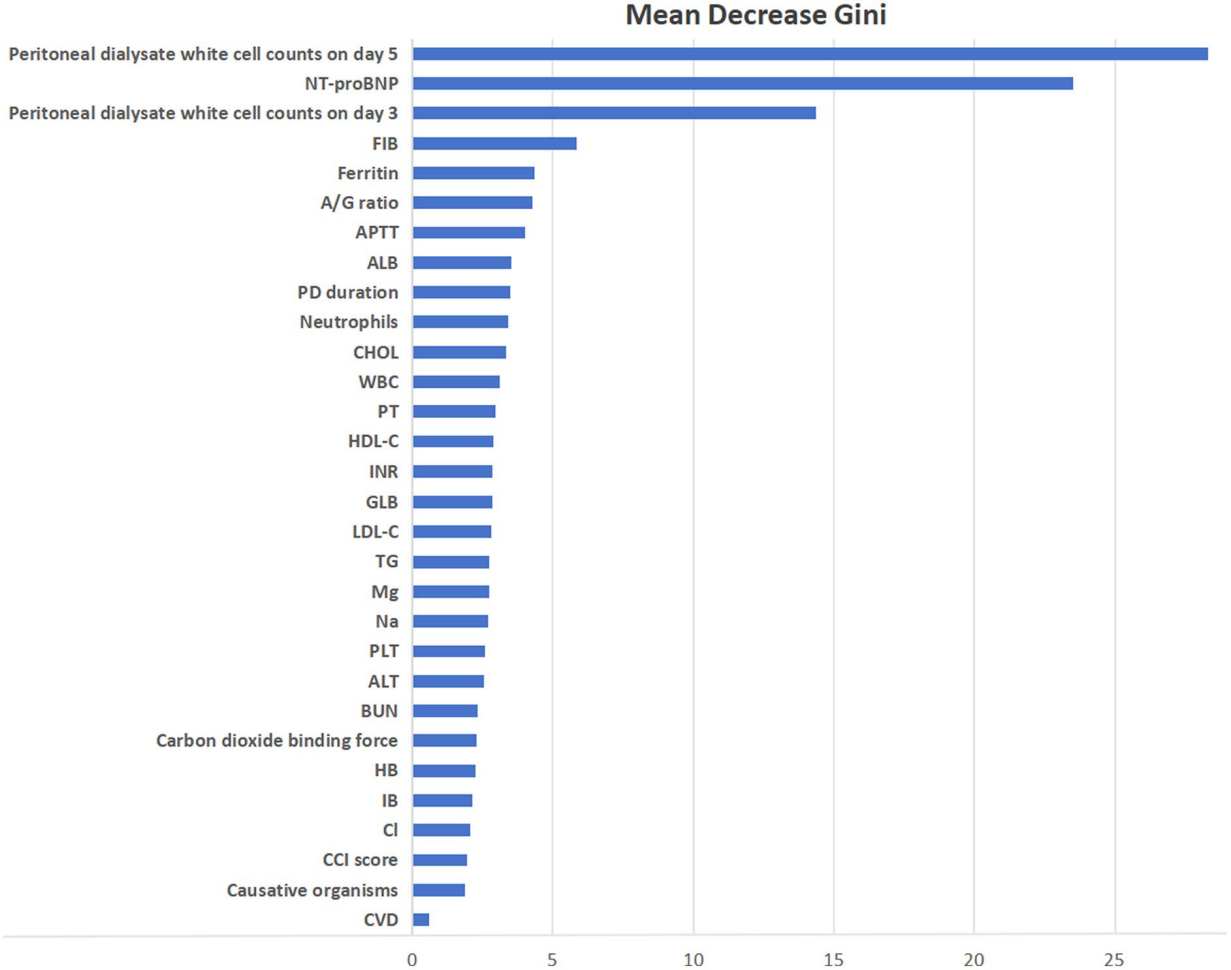
Ranking the importance of variables based on RF model.

What’s more, we assessed the importance of variables based on mean decrease Gini index in the RF prediction model (Figure 5). Mean decrease Gini indicates the importance of the independent variables to the dependent variable, which refers to the total decrease of Gini from splitting on the variable averaged over all trees. And the top 5 predictors for technique failure were peritoneal dialysate white cell count on day 5, NT-proBNP, peritoneal dialysate white cell count on day 3, FIB and ferritin.

## Discussion

Our study is the first to develop the prediction models by multiple ML algorithms and then select the best one for technique failure in PDAP. The RF model could accurately predict the technique failure of PDAP patients, which had the most excellent predictive performance among the five prediction models. And RF model could assess the importance of predictors associated with technique failure intuitively.

Previous studies have developed prediction models for the treatment failure in PDAP patients only using LR. One multicenter and retrospective cohort study conducted in Thailand developed a risk prediction tool for peritonitis associated technique failure based on multivariate LR (15). Meng et al. established a nomogram for the prediction of peritonitis cure by using multivariate LR in PDAP patients. Although, the prediction nomogram model was intuitive and applicable to clinical practice, its C-statistic value was 0.756 (16). Another two single-center studies also only used LR analysis to established prediction models, and they lacked independent datasets to validate their prediction performance (17, 18).

Unlike the previous studies only using LR to construct models, our study used 5 ML algorithms and we found that RF model was more suitable for predicting technique failure in PDAP patients than the other four prediction models. Due to the different methodological analysis of machine learning algorithms, their performance varies when dealing with the same data or problems. Random forest performs well in handling complex data or multivariate problems. Random forest is an ensemble learning algorithm, which is a classifier containing multiple decision trees (23). The final categories are determined according to the voting of multiple decision trees. Random forest can select samples or variables randomly to avoid overfitting. At the same time, random forest has strong adaptability to data, which can handle datasets containing a large number of variables and balance the errors generated by unbalanced datasets. What’s more, random forest can still maintain high accuracy even if some features are lost (24). However, LR requires the assumption of linear relationships between variables, and variable transformations are required for nonlinear data, which is not always applicable to all variables in clinic (25). Furthermore, LR is prone to insufficient fitting and poor classification accuracy (26).

The previous studies did not incorporate some important and readily available variables, such as NT-proBNP, peritoneal dialysate white cell count on day 3 or day 5, FIB, or ferritin. We included all possible variables to avoid ignoring important variables in our prediction models. Our study demonstrated that NT-proBNP, FIB and ferritin were important predictors for technique failure in PDAP patients. When selecting variables, we conducted cross-validation to try to avoid overfitting or underfitting issues, which improved the generalization ability of prediction models.

Our study found several important predictors of technique failure. In the RF prediction model, the peritoneal dialysate white cell count on day 5 was the most important predictor, which was consistent with previous studies. A previous risk-scoring scheme demonstrated that peritoneal dialysate white cell count on day 5 was the strongest predictor, which was responsible for 88% of their predictive model (15). The retrospective study included 565 peritonitis episodes and found that the predictor of peritoneal dialysate white count >100/uL for at least 5 days showed a significant association with treatment failure [odds ratio (OR) 7.38; 95% confidence interval (CI) 3.38 to 16.1] (27). Another retrospective study of 399 peritonitis episodes, Krishnan et al. reported that the nonresolution rate was significantly higher in patients with the peritoneal dialysate cell counts exceeded 100/uL for more than 5 days (28). The peritoneal dialysate white cell count on day 3 was another important predictor of technique failure, which has been previously recognized (15, 27). Comparing to the peritoneal dialysate white cell count on day 3, the cell count on day 5 might reflect the response of treatment better (15). But the peritoneal dialysate white cell count on day 3 might be more valuable to the clinicians who desired to refine the treatment strategy during the early course of PDAP (27). We recommend the peritoneal dialysate white cell count should be measured on a routine basis, especially on day 5 and day 3.

NT-proBNP was a significant predictor of technique failure which was not reported in previous studies about PDAP. There were several studies demonstrated that elevated NT-proBNP was associated with adverse outcomes in PD patients. The Adequacy of Peritoneal Dialysis in Mexico (ADEMEX) study enrolled 965 CAPD patients and found that NT-proBNP was independently highly predictive of overall survival and cardiovascular mortality (29). Two prospective cohort studies also reported that NT-proBNP is an important risk predictor of cardiovascular congestion, mortality, and adverse cardiovascular outcomes (30), and was independently predictive of an increased risk of technique failure in maintenance PD patients (31). During PDAP, the increased permeability of peritoneal membrane due to inflammation might result in decreased ultrafiltration and the subsequent volume overload and elevation of NT-proBNP. Volume overload and even congestive heart failure demand emergent hemodialysis, which may contribute to the technique failure in PDAP. This could be partially avoided by the use of icodextrin PD fluid. But icodextrin PD fluid was just available in China in recent 2 years. We still recommend it is necessary to monitor NT-proBNP regularly during PDAP and explore the relationship between NT-proBNP and fluid status in PDAP patients in the future.

With the help of prediction model, we could accurately distinguish the high-risk patients prone to technique failure in PDAP. For the high-risk patients, we suggest a closer assessment of relevant predictors, application of metagenomic next-generation sequencing (mNGS) to identify the causative organisms timely (32), appropriate adjustment of antibiotic regimens, or considering PD catheter removal or transfer to HD promptly. For the low-risk patients, outpatient treatment may be feasible, which could effectively decrease the hospitalization time and health care costs, but close monitoring is still necessary.

There were some limitations in our study. Firstly, the cohort only included Chinese patients of a single PD center and the laboratory data was affected by geography and demographics. Our prediction models were internally valid, but we lacked external validation. Therefore, the generalizabilities of the prediction models were limited. Secondly, some variables such as IL-6, hs-CRP or PCT were excluded due to high missing rates. These variables may be potential predictors for technique failure, which can be explored in future. Thirdly, this was a retrospective study and might have selection bias. Several novel biomarkers showing good predictive performance of adverse outcomes in PDAP patients, which could be incorporated into the construction of prediction models in future (33, 34). Multicenter and large data are required for the external validation and to evaluate the clinical utility of the prediction models.

## 5 Conclusion

In conclusion, our study constructed a reliable prediction model based on RF algorithm to predict the risk of technique failure for PDAP patients. The RF model demonstrated the best predictive performance and highest accuracy, which could accurately predict the technique failure and assist in clinical decision-making.

## Data availability statement

The raw data supporting the conclusions of this article will be made available by the authors, without undue reservation.

## Ethics statement

The studies involving humans were approved by Medical Ethics Committee of West China Hospital of Sichuan University, Sichuan, China. The studies were conducted in accordance with the local legislation and institutional requirements. The participants provided their written informed consent to participate in this study.

## Author contributions

ZZ: Writing – original draft, Writing – review & editing. QX: Writing – original draft. XZ: Writing – original draft. NM: Writing – review & editing. LP: Writing – review & editing. YT: Writing – review & editing. ZL: Writing – original draft, Writing – review & editing.

## Figures and Tables

**Table:** 

PDAP	Peritoneal dialysis associated peritonitis
PD	Peritoneal dialysis
ML	Machine learning
RF	Random forest
LASSO	The least absolute shrinkage and selection operator
KNN	k nearest neighbor
LR	Logistic regression
AUC	Area under curve
ESRD	End-stage renal disease
COVID-19	Coronavirus disease 2019
HD	Hemodialysis
ISPD	International society for peritoneal dialysis
SBP	Systolic blood pressure
DBP	Diastolic blood pressure
BMI	Body mass index
DM	Diabetes mellitus
CVDs	Cardiovascular diseases
CCI	Charlson comorbidity index score
HB	Hemoglobin
WBC	White blood cell
ALB	Serum albumin
SCr	Serum creatinine
BUN	Blood urea nitrogen
UA	Uric acid
eGFR	Estimated glomerular filtration rate
TG	Triglycerides
HDL-C	High-density lipoprotein cholesterol
LDL-C	Low-density lipoprotein cholesterol
K	Serum potassium
Ca	Serum calcium
NT-proBNP	N-terminal pro-brain natriuretic peptide
hs-CRP	High-sensitive C-reactive protein
FIB	Fibrinogen
CAPD	Continuous ambulatory peritoneal dialysis
APD	Automated peritoneal dialysis
SD	Standard deviation
IQR	Interquartile range
ROC	Receiver operating characteristic curves
OR	Odds ratio
CI	Confidence interval
ADEMEX	Adequacy of peritoneal dialysis in Mexico
mNGS	Metagenomic next-generation sequencing
